# A systemic review of barriers to accessing paediatric eye care services in African countries

**DOI:** 10.4314/ahs.v21i4.47

**Published:** 2021-12

**Authors:** Saif Hassan Alrasheed

**Affiliations:** 1 Department of Optometry, College of Applied Medical Sciences, Qassim University, Saudi Arabia; 2 Faculty of Optometry and Visual Sciences, Department of Binocular vision Al-Neelain University, Khartoum, Sudan

**Keywords:** Paediatric eye care, Africa, availability, accessibility, affordability, visual impairment, refractive errors

## Abstract

**Background:**

Global estimate reported that 1.4 million children are blind of which three-quarters live in developing countries. Childhood Visual Impairment is a major public health problem globally especially in rural areas of developing countries.

**Objective:**

To review barriers to accessing paediatric eye care services in African countries

**Methods:**

The studies in this review were searched in online databases (PubMed, Web of Sciences, ProQuest, Scopus, Google Scholar, African Index Medicus and Medline) for studies published between January 2000 and April 2020. The articles included in this review, which was conducted in Africa to assess the barriers for accessing paediatric eye care services with regards availability, accessibility, affordability, socio cultural barriers of parents/caregivers and community.

**Results:**

Of 22 705 articles screened, the study found 29 publications from 10 African countries which met the inclusion criteria. The main barriers were non-availability, non-accessibility, and non-affordability of paediatric eye care services. The studies reviewed revealed that there are other factors affecting the utilization of paediatric eye services which include the primary health system, geographic barriers, health beliefs, perception of parents; lack of knowledge, attitudes and practices about paediatric eye care. Furthermore, environmental, demographic barriers and socio-economic status has negative impact on accessing paediatric eye care services in African counties.

**Conclusion:**

The main barriers to accessing paediatric eye care services in Africa were affordability, accessibility and availability. There is therefore a need for all relevant stakeholders to play a significant role in addressing barriers to child eye care in African countries.

## Introduction

Global estimates indicate that there are around 19 million visually impaired children worldwide, of these, 1.4 million are blind, and 17.5 million have low vision and most of them are found in developing nations^1^. Global initiative for avoidable blindness reported that the major barrier to accessing paediatric eye care in Africa was unavailability of primary eye health services^2^,^3^. The World Health Organization (WHO) reported that most of paediatric visual impairment (VI) is treatable by early intervention at primary, secondary, and tertiary level^2^. Of concern, paediatric eye care services in many African countries are not prioritised on the public health agenda regardless of legislation and guidelines being available, therefore highlighting the fact that barriers to policy development such as politics, poor performing economies and poor health financing systems continue to persists in these countries^4^–^33^. Although, VI among children is low compared to adults, it has serious significant negative impact on the lifespan of the child with an estimate of 60% of children dying within one year of becoming blind^3^,^4^.

Barriers to eye care are broadly defined as factors that affect people from accessing services such as healthcare, which negatively influences both services deliver and access. It is expected that without intervention programs to prevent avoidable childhood blindness; the global loss will be more than US$ 110 billion by the year 2020 5. Children in most instances fail to report their visual limitations while teachers, parents and guardians find it difficult to identify them constituting a barrier to early intervention 4 However, paediatric eye care services in the most African countries are inadequate in terms of facilities, equipment, resources, and skilled professionals. For instance, there are only 26 paediatric eye-care centres in sub-Saharan African countries serving 787 million with a ratio of one paediatric eye care centre for 30.3 million children. Available workforce in the African continent is reported to be inadequate and most countries have far less than the optometrist to population required as per the guidelines of the WHO^5^–^9^. Moreover, the International Council of Ophthalmologists indicated that two-third of all ophthalmologists worldwide are from high-income countries while the rest of world share the remaining, resulting in inadequate paediatric eye care providers in African countries^10^,^11^. WHO recommended that at the minimum, there should be one paediatric eye care centre per ten million populations 12,13,14. In some countries where paediatric eye services exist, they are underutilised because of many existing barriers such as socio economic status, cultural beliefs, unawareness of signs and symptoms^1^–^5^ Therefore, barriers leading to poor utilisation of paediatric eye care services in the African region were reviewed in order to address them.

## Methodology

### Systematic search

The systematic review in this article included all the studies conducted to assess the barriers to accessing paediatric eye care services in African countries from January 2000 to April 2020. Eligible articles were those that: (1) assessed financial barriers (insurance, income, employment status) (2) educational brriers (inability to obtain, influence and perceptions, understanding and using information). The accessibility to paediatric eye care services was assessed by exploring five dimensions: 1) availability and accommodation; 2) accessibility; 3) affordability; 4) acceptability; 5) and appropriateness. In order for these dimensions to ensure accessibly, they need to interact (these being: (1) ability to seek, (2) ability to perceive, (3) ability to reach, (4) ability to pay, and (5) ability to engage). The affordability indicator was used in this study to describe the ability of community to pay for cost of paediatric eye care. While the term of accessibility is defined as the ability of community to receive paediatric eye care from primary to tertiary levels. Appropriateness in the current study used to describe paediatric eye care in line with evidence-based or consensus-based.

The studies included cross-sectional epidemiology survey, prospective observational studies, qualitative and quantitative studies. The articles were excluded if (1) they were not conducted in African (2) studies that did not assess barriers to accessing childhood eye care services. The study also excluded, conference paper, meeting abstract, editorial discussion and studies without basic data collection. The articles included in this review were searched online databases Web of sciences, PubMed, ProQuest, Scopus, Google Scholar, African Index Medicus and Medline using the main keywords of search in Table 1.

**Table 1:** Search keywords used for systemic review of barriers for accessing childhood eye care services in African countries

**Paediatric eye care services**
Visual Impairment/ Vision Impairment* Refractive Error/ Myopia* Avoidable blindness/ Ocular diseases* Strabismus/ Amblyopia* Eye care services/ Visually impaired*
**Barriers for accessing paediatric eye care**
Accessibility of paediatric eye care* Health seeking/ Eye care seeking* affordability/ cost* Access/ Utilize* Availability/ Easy to get eye care* Attitude/ Perceptions* Spectacles wear* Parents/ Caregivers*

## Results

**Figure 2:**
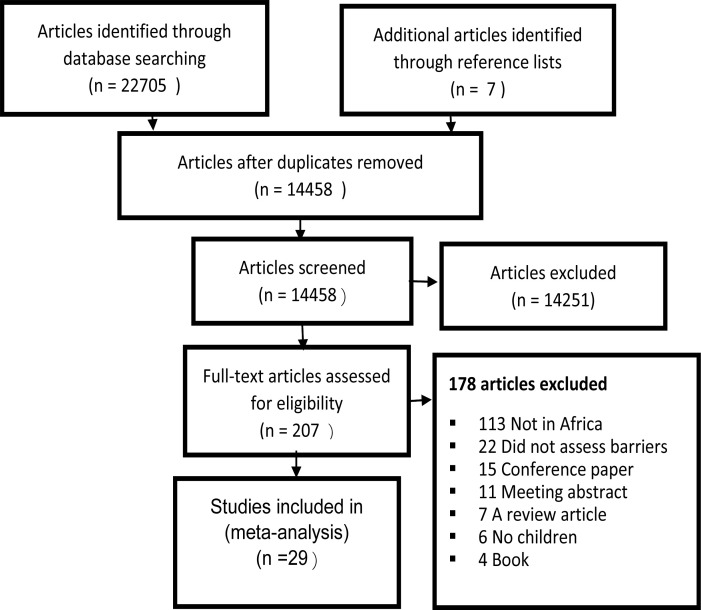
Flowchart used for the systematic review of barriers to accessing paediatric eye care services in African countries

Of the 22 705 articles initially captured, the study found 29 publications from 10 African countries that met the inclusion criteria (Fig. 2)^12^–^63^. The studies reviewed mostly reported that the main barriers were non-availability, non-accessibility, and non-affordability of eye care services in addition to paediatric eye care not being a priority agenda in many Africa health systems^6^–^32^. The studies reviewed revealed that there are other factors affecting the utilization of paediatric eye services which include the public health system, geographic barriers, health beliefs, perceptions of parents; lack of knowledge, attitudes and practices about childhood eye care. Furthermore, environmental, demographic barriers and socio-economic status has negative impact on accessing paediatric eye care services in African counties^32^–^62^.

## Discussion

### Factors affecting utilization of paediatric eye care services in African countries

Several authors 6–22 highlighted three main reasons for the high prevalence of childhood VI in Africa which include non-availability, non-accessibility, and non-affordability of eye care services. Studies included in this review acknowledge the fact that even in countries where facilities are available, accessible and affordable barriers such as lack of knowledge, misconception of the consequences of a childhood eye disease and misunderstandings on who to consult for treatment therefore impedes proper utilisation. Moreover, demographics, geographical landscape, and socio-economic status and environmental factors constitute barriers to accessing available and affordable childhood eye care services in African 8–32.

### Availability, accessibility and affordability of paediatric eye care services

Socio-economic factors such as income, employment, and insurance were found to be having positive association with use of the eye care services among communities^29^,^37^,^38^. All the highlighted educational and socio-economic status barriers have negative impact on available and accessible childhood eye care services among African poor community.

### Availability of paediatric eye care services

The availability and distribution of eye care professionals is far less in Africa as compared to developed countries with nine times more eye care professionals per million population8-12. Within African countries, availability of paediatric eye services varies from region to region, from district to district and even from one community to another^8^–^32^. Furthermore, the optometrist to population ratio remains low due to non-prioritisation and/or absence of training institutions within the African continent. The situation is dire even in countries that are reported to have training institutions, which therefore requires policy formulation/review then amendment in order to rectify the inadequate numbers, skewedness, lack of infrastructure, improve knowledge base within the respective countries^9^. The African child strategy forum also reported that eye care services were inadequate and skewed in distribution which impacts negatively on accessibility to these services. 5 In support, studies from Cameroon5′9, Ethiopia^5^, Sudan and South Africa^5^,^7^ acknowledged the skewedness of ophthalmologists distribution being mainly at urban areas as well as few that are working in the public service^5^–^20^. This worsens the situation particularly among those living in remote areas as they remain sidelined with poor access to eye care services.

School health system in Swaziland does not function optimally due to the lack of human resource, with only two ophthalmic nurses to attend to referred children in the entire country15. The authors^14^,^15^,^55^ cited that the reasons of unavailability of paediatric eye care services could be due to poor design/formulation of policy which results in poor functioning health systems in most of African countries that fails to inform eye care policy plans in the respective countries. The levels of care in particular the primary level, therefore needs to be reconfigured in order to ensure adequate distributions of these services.

### Accessibility to paediatric eye care services

Poor accessibility to eye care services is a major factor contributing to the high prevalence of childhood blindness in Africa^7^–^33^. The common identified barriers for accessing paediatric eye care services among African communities were long distance travelled to appropriate eye care facilities and poor road infrastructure^13^–^60^. A significant portion of the population (40%) of Sudan have no access to health services located within 5km radius particularly for those residing in rural areas18. Similarly, in Swaziland children with eye problems were referred to another region that offers ophthalmic services, due to the unavailability of a paediatric eye health facility in their region^15^. Consequently, available services in urban areas end up being underutilised while a significant portion of children live with avoidable and treatable eye conditions^19^–^23^. Nigerian parents expressed that getting time off work, long waiting period at clinic were barriers for accessing paediatric eye care services^20^. The report highlighted that logistics barriers had significant effect on approachability to paediatric eye care and recommended that these obstacles should be considered when planning eye care programs^20^.

A survey conducted in Africa to assess tertiary paediatric eye care services revealed that there are 21 centres located in 10 out of 42 countries^13^. The authors13 suggested that strengthening existing paediatric eye care services in Africa would need investment in manpower (childhood blindness coordinators, eye care professionals and others stakeholders). In addition, programs to recognise and refer children requiring services and mechanisms to support the reasonably high price of providing this service. Another study in Malawi reported that long distance travelled to health facilities lead to decrease acceptance of free childhood eye services. The study concluded that providing eye care services free of cost may not be adequate for the poorest people in rural Africa^60^.

The environment around the people may affect their access to paediatric eye care services. These barriers include difficulties with transportation, great distances to a health centre, long appointment waiting time and other barriers affecting access to childhood eye care, these obstacles were cited by several authors^7^,^13^,^20^,^24^,^39^ has huge impact on availability and accessibility to paediatric eye care among African communities. A study conducted in South Africa by Mashige, et al. to determine the barriers to access eye care services, 29.1% of responders stated that they were unable to get treatment due to long waiting times^26^. Another study conducted in Sudan parents were cited lack of transportation and faraway of eye care centre as barriers for accessing childhood eye care services^13^. Parents in Nigeria were reported long waiting periods at the clinic as barriers for accessing a child eye care^20^. This could be due to most people during seeking eye care skip levels and present to higher levels without following referral protocols and turned away sometimes. Furthermore, the poor road infrastructure/poor transport system has been documented as contributing to poor accessibility in many African countries.

### Affordability of paediatric eye care services

The income level of people and the cost of paediatric eye care services influence affordability of eye care services in Africa 21–63. Poverty is a major problem specifically in poor African nations, therefore, the people are unable to afford the cost of paediatric eye care services, and results in conditions that could have been treated at an early stage being left untreated and may cause visual impairment^7^–^22^. Africa with approximately 10 percent of world's population and carry 19 percent of the world's blindness, this could be due to a combination of factors such as poverty, lack of education and inadequate eye care services^22^–^63^. Nigeria is second economic power in Africa^20^, however the blindness in Nigeria is associated with poverty, in part reflecting lower access to services^23^. Studies have reported that cost is a major barrier in accessing paediatric eye care in Nigeria, Sudan and Ethiopia^20^,^24^,^25^. They recommended that efforts were needed to create mechanisms that could bridge communities and eye care facilities. Mashige, et al. 26 conducted a study to determine the utilization of eye care services in South Africa they revealed that 80.5% of participants received the recommended treatment for their eye conditions, however 36.4% of respondents reported that they did not get the management due to affordability. South African considerd the leading economy in Africa and also the cost of eye care was cited as main barriers for accessing eye care services. A study conducted in Nigeria to assess the reasons for rescheduling of surgery for paediatric cataract in a tertiary hospital the study showed that the main reason was financial restrictions^35^. The main restriction preventing children in Lagos, Nigeria from using spectacles was affordability58. In Tanzania main barriers for accessing spectacle among children was cost^55^. So that, when individuals have financial difficulties, their priorities may not be on the prevention and treatment of vision impairment and people of low-income might not have health insurance for covering eye care. In Sudan72.6% of participants stated that their health insurance did not cover eye care services^24^. From above mentioned in reviewed studies paediatric eye care services are not supported in African countries and the popultion still suffer to afford as this could be due to prioritization of other health care illnesses than eye health particularly in the public sector. WHO African region reported that regardless of progress improvement of health in many African countries are still on average far from achieving their health financing goals such as the Abuja target of allocating 15% of government budgets to health. Majority of African countries out-of-pocket costs are still higher than 40% of the total health expenditure. This figure reflected that in general the health financing systems in Africa are weak and do not ensure sustainable progression and equity in the way funds are collected and invested^64^.

### Effect of demographic, social and cultural factors on utilization of paediatric eye care

These aspects sometime overlap and multi-factorial solutions are needed to address the problem. There are many underlying public health principles that affect availability, accessibility, and affordability of the childhood eye care services in Africa. The health care system needs to be organized to provide primary eye care as an entry point into the health care system. For example, paediatric eye care as an entry point into the primary care system. The childhood eye care should be provided at all health care level to cover prevention and treatment; open an effective communities' communication and eye health knowledge should exist between health care providers and the patients; eye care professionals should use best practices with patients and patients should assume responsibility for their health; and health care access should be provided across the entire lifespan, and no individual should be excluded from health care despite any conditions^4^–^17^. The demographic, such as gender, age, education, socio-economic status, social and cultural factors (human behaviour, thoughts, beliefs, attitudes, knowledge and perceptions) influence the utilization of paediatric eye care services in Africa some of these factors discussed below 4,24–62.

### Knowledge, Attitude and Perception(KAP) barriers

The educational barriers affect most of population and health care providers; the high level of education associated with positive use of eye care services; people with lower education levels may experience lack of knowledge about the importance of regular eye examinations and find it difficult to know the signs and symptoms of eye disorders4. Many people believe that if they had an eye disease they would have symptoms. The silent eye conditions such as glaucoma and amblyopia are usually difficult to detect even among knowledgeable and well-educated parents hence the study from Swaziland may have found no significant difference.34 WHO reported that major cause of global childhood vision impairment is uncorrected refractive error and estimates that 80% of vision impairment is avoidable by early diagnosis and management.1 Therefore, improved awareness of the community about paediatric eye care is very important in developing countries and could help in timely diagnosis and management of eye conditions. In many African countries awareness about childhood eye problem is low resulting in high prevalence of preventable childhood visual impairment^12^–^48^. In Swaziland majority of eye health professionals reported unawareness of available services by parents as the most common barrier to accessing eye care services^39^. Furthermore, Sukati, et al.^34^ stated that 53.1% of parents have no knowledge about paediatric eye conditions. Majority of parents in Nigeria reported that they would look for eye examination for their children only when the child has some eye problems^40^. Another study conducted among Nigerian school-aged children to identify eye health myths and misconceptions concluded that most of the children do not have basic information about eye care^41^. A study among Kenyan high school students to determine the Knowledge, Attitudes and Practices (KAP) towards refractive error revealed that almost 39% of the students have never had an eye examination^42^. Moreover, study carried out among Ethiopian parents concluded that the levels of knowledge towards childhood blindness prevention and treatment were low. Therefore, educational interventions to increase the knowledge on treatment and prevention of childhood blindness are important to reducing the high prevalence of visual impairment in Africa^43^. A study conducted in Kenya to assess the KAP of eye disease in children among pediatricians, revealed that the pediatricians had poor level of knowledge on pediatric eye diseases^44^. In another study conducted among Nigerian guardians to assess attitude towards childhood eye care. The result indicated the negative implications of neglecting eye diseases and use of self and harmful traditional eye-medications by the parents need to be eliminated by suitable eye health educational intervention^45^. Also ignorance of refractive error as barriers reported among Nigerian students58. However, in Ghana46 almost 76% of mothers had good knowledge about childhood blindness, the authors concluded that maternal knowledge about paediatric eye care was high. Nevertheless, intervention educational program that concentrate on increasing level of parental awareness of childhood visual impairment are needed^46^. A study in Ethiopia to assess the knowledge, attitude and associated factors towards refractive error among primary school teachers concluded that Knowledge and attitude towards refractive error was low among teachers and recommended that eye health educational program needed for educators^47^. About 94.3% of Nigerian parents repoted that they commonly use self-medication and local remedies for treatment of conjunctivitis48. About 54% of the respondents visiting a tertiary eye clinic in southern Nigeria had no knowledge of the treatment of the strabismus^53^. Therefore, programs to increase awareness of causes of eye diseases and dangerous effects of self-medication are necessary for the community^48^. Parents in Tanzania reported that cataract mainly related to old age, the majority of parents expressed astonishment at the diagnosis, as they were not aware a child could have cataract^61^. Government and NGOs in Africa should deliver childhood eye care education through the public media, radio, television, social media and newspaper, to improve awareness of community about childhood eye care services and understand the importance of early diagnosis and treatment of paediatric eye condition.

Several authors 42,49,50,51,52 reported that the attitudes and perceptions towards eye care and spectacles for correcting refractive error were the main barriers for accessing pediatric eye care in developing countries. In many African communities' parents believe that young children should not wear spectacles, regarding to their perception strabismus was considered as irreversible eye condition. Alrasheed, et al 24 revealed that about 45.6% of parents from South Darfur State of Sudan believe that strabismus is an untreatable eye condition. Moreover, about 14% of the parents showed that local remedies of strabismus are more effective than the treatment in the hospital. Whilst other parents believed that childhood eye diseases, does not require treatment and could resolve spontaneously. In Nigeria, about 21.33% of parents reported that they ignored looking for healthcare for childhood eye diseases and almost 9.26% and 7.76% reported that they use self-medication and local remedies, respectively for treatment of pediatric eye condition^45^. Reasons for using self-medication/ local remedies in treatment of childhood eye problems could be due to inadequate paediatric eye care services, higher cost, and cultural beliefs, combined with low levels of education. In Sudan, learners believed that wearing spectacles affected their opportunities for education, employment and marriage^51^. Alrasheed, et al 51 suggested that paediatric eye health education packages should be broadcast through the public media to promote attitudes, perception and benefits about spectacle wear and eye care.

Parents in Nigeria stated that using spectacles would damage their children's eyes^45^. While others believed that glasses were meant for old people^53^. Parents in Sudan reported that the disadvantages of wearing spectacles could lead to development of poor eye sight and spectacle wear had psychological impact, particularly among females^51^,^62^. Students in Tanzania were happy with appearance of their eye glasses and the valuable effect on their vision, but their parents were concern about safety of spectacle wear^54^. Consequently, health education and enlightenment strategies should be put in place by professional bodies and the government to increase parent's attitude and perception towards childhood eye care services^40^. A study conducted among visually impaired children in South Africa to know disability related distress due to uncorrected refractive error (URE) indicated that vision impairment due to URE can cause distress in different domains in children. The study recommended that distresses due to URE should be taken into consideration when developing paediatric eye care strategy^56^. Study conducted among schoolaged children in Egypt revealed that visual impairment has impact on self-confidence and quality of life and recommended that it is critical for planning and implementing of a psychological counseling platform for children with visual impairment to improve their emotional condition^59^. As highlighted above from reviewed articles the fear, stigma and bad perceptions about paediatric eye care services are commonly experienced among Africa communities. Therefore, paediatric eye care education platforms should be delivered through the public media to encourage awareness and benefits about eye care.

## Conclusion

The main barriers to accessing paediatric eye care services in Africa were non-availability, non-accessibility, and non-affordability, lack of knowledge, negative attitudes of parents and primary health system. In terms of accessibility to the health care, previous studies, indicate that a high proportion health services are concentrated in the capital cities. Furthermore, there was a huge disparity in the distribution of healthcare providers in the various States in African nations. Hence, paediatric eye care plan with school-based eye-care interventions could have great effect to reduce barriers to accessing eye care. Teachers, nurses and trained personals could help to provide school vision screenings, particularly in some African countries where there is a marked lack of eye care professionals. The studies reviewed revealed that there are others factors affecting the utilization of paediatric eye services which included the public health system and the demographic characteristics of population such as educational level, socio-economic status, misconception as well as the environment around the people. Barriers to accessing paediatric eye care services are broad and some are interrelated, it is better to address when interventions are applied in combination, as no single aspect addresses all barriers in order for eye health services to be available, accessible, appropriate and acceptable. Strategy is needed for improving knowledge, attitudes and practices of the key stakeholders such as parents and teachers could play a crucial role in addressing these barriers. There is therefore a need for all relevant stakeholders to play a significant role in addressing barriers to child eye care in African countries.

## Figures and Tables

**Figure 1:**
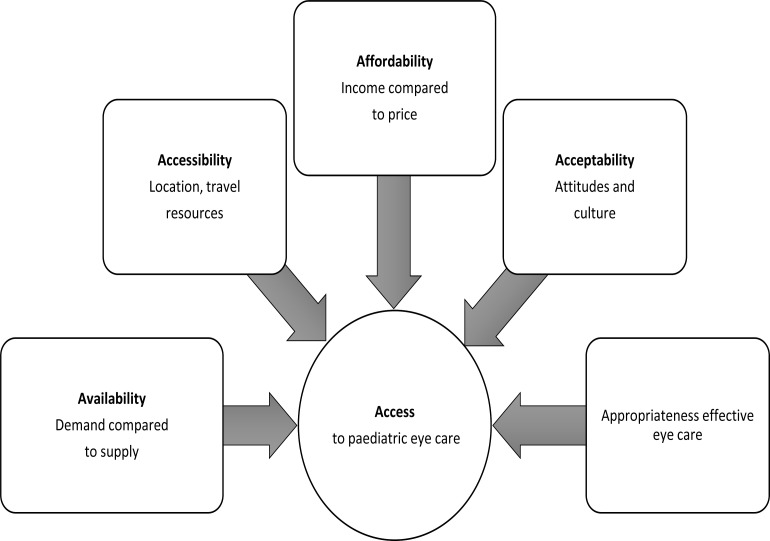
Dimensions of accessibility

**Table 2:** Studies identified in the systemic review about barriers to accessing paediatric eye care services in African countries

Study	Country	Study design	Study sample	Barriers identified
Agarwal *et al.*, 2010^13^	Multi-country	Cross-sectional (prospective)	27 health tertiary facilities	Availability, accessibility and affordability
Alrasheed et al., 2018^14^	Sudan	Qualitative (Delphi)	18 eye-care providers	Availability, accessibility and affordability
Sukati *et al.*, 2018^15^	Swaziland	Mixed	9 eye health facilities	Availability, accessibility and affordability
Ebeigbe 2018^20^	Nigeria	Qualitative (narrative)	35 parents and 10 eye- care practitioners	Parents behaviour
Alrasheed, *et al.* 2016^24^	Sudan	Quantitative and qualitative methods	387 Pupils and 47 parents	KAP of the students and their parents
Sukati, *et al* 20018 34	Swaziland	Quantitative	173 parents	KAP of parents
Ugalahi, *et al* 2020 35	Nigeria	Quantitative	164 children	affordability
Chan, *et al* 2017^36^	Tanzania	Quantitative-(Prospective)	1051 participants	Parents behaviour
Sukati, *et al* 2019^39^	Swaziland	Quantitative	15 public eye health professionals	Availability and accessibility of child eye care services
Amiebenomo, *et* *al* 2016^40^	Nigeria	Quantitative	468 parents	KAP parents
Oguego, *et al* 2018^41^	Nigeria	Quantitative	833 Students	Eye health misconceptions
Nyamai, *et al* 2016^42^	Kenya	Quantitative	1390 students	KAP of students towards RE
Belaynew, *et al* 2014^43^	Ethiopia	Quantitative	1315 households	KAP of community towards childhood blindness
Wanyama 2013^44^	Kenya	Descriptive	125 pediatricians	KAP of child eye disease among pediatricians
Ayanniyi, *et al* 2010^45^	Nigeria	Quantitative	1,393 guardians	Guardians' attitude towards eye health
Kumah, *et al* 2017^46^	Ghana	Hospital-based cross-sectional	100 mothers	Knowledge of paediatric blindness among mothers
Alemayehu, *et al* 2018^47^	Ethiopia	Institution-based cross-sectional	565 primary school teachers	KAP and associated factors among teachers about RE
Ebeigbe, *et al* 2017^48^	Nigeria	Qualitative	35 parents	Parents knowledge about their children's eye problems
Alrasheed *et al* 2018^51^	Sudan	School-based cross-sectional	387 Students and 47 parents	Attitudes and perceptions towards spectacle wear
Isawumi, *et al* 2014^53^	Nigeria	Descriptive cross- sectional	405 respondents	Perceptions, towards treatment of childhood strabismus
Odedra, *et al* 2008^55^	Tanzania	Qualitative	8 focus groups discussion	Barriers to spectacle use
Mafwiri, *et al* 2014^56^	Tanzania	Comparison study	45 Clinical Officers	Accessibility child eye health
Chan, *et al* 2020 57	South Africa	Qualitative	93 Children	Attitude and perceptions
Faderin and Ajaiyeoba 2001 58	Nigeria	Quantitative	919 pupils	Barriers to acceptance of wearing glasses
Megbelayin 2013 59	Nigeria	Quantitative	1,241 pupils	Barriers to acceptance of prescribed spectacles
Kotb, *et al* 2010 60	Egypt	Descriptive	100 children	Attitudes and perception
Schulze *et al* 2014 61	Malawi	Qualitative study	58 parents	Barriers to acceptance of free pediatric cataract surgery
Shirima *et al* 2006 62	Tanzania	Qualitative study	117 parents	Barriers of pediatric cataract surgery among parents
Alrasheed 2020^63^	Sudan	Quantitative	80 schoolteachers	Teachers' Perspectives about childhood eye care

## Declaration

The authors declare that they have no financial or personal relationships that may have inappropriately influenced them in writing this article.
